# Assessment of a Pilot Program for Remote Support on Mental Health for Young Physicians in Rural Settings in Peru: Mixed Methods Study

**DOI:** 10.2196/54005

**Published:** 2024-09-10

**Authors:** Kelly De la Cruz-Torralva, Stefan Escobar-Agreda, Pedro Riega López, James Amaro, C Mahony Reategui-Rivera, Leonardo Rojas-Mezarina

**Affiliations:** 1 Unidad de Telesalud Facultad de Medicina Universidad Nacional Mayor de San Marcos Cercado de Lima Peru; 2 Department of Biomedical Informatics University of Utah Salt Lake City, UT United States

**Keywords:** telemedicine, screening, treatment, mental health, suicide, depression, anxiety, alcoholism, physicians, rural areas, Peru

## Abstract

**Background:**

Telemedicine-based interventions show promise in addressing mental health issues among rural populations, yet evidence regarding their impact among the health care workforce in these contexts remains limited.

**Objective:**

This study aimed to evaluate the characteristics and the responses and perceptions of recently graduated physicians who work in rural areas of Peru as part of the Servicio Rural Urbano Marginal en Salud (Rural-Urban Marginal Health Service [SERUMS], in Spanish) toward a telehealth intervention to provide remote orientation and accompaniment in mental health.

**Methods:**

A mixed methods study was carried out involving physicians who graduated from the Universidad Nacional Mayor de San Marcos and participated in the Mental Health Accompaniment Program (MHAP) from August 2022 to February 2023. This program included the assessment of mental health conditions via online forms, the dissemination of informational materials through a website, and, for those with moderate or high levels of mental health issues, the provision of personalized follow-up by trained personnel. Quantitative analysis explored the mental health issues identified among physicians, while qualitative analysis, using semistructured interviews, examined their perceptions of the services provided.

**Results:**

Of 75 physicians initially enrolled to the MHAP, 30 (41.6%) opted to undergo assessment and use the services. The average age of the participants was 26.8 (SD 1.9) years, with 17 (56.7%) being female. About 11 (36.7%) reported have current or previous mental health issues, 17 (56.7%) indicating some level of depression, 14 (46.7%) indicated some level of anxiety, 5 (16.6%) presenting a suicidal risk, and 2 (6.7%) attempted suicide during the program. Physicians who did not use the program services reported a lack of advertising and related information, reliance on personal mental health resources, or neglect of symptoms. Those who used the program expressed a positive perception regarding the services, including evaluation and follow-up, although some faced challenges accessing the website.

**Conclusions:**

The MHAP has been effective in identifying and managing mental health problems among SERUMS physicians in rural Peru, although it faced challenges related to access and participation. The importance of mental health interventions in this context is highlighted, with recommendations to improve accessibility and promote self-care among participants.

## Introduction

In Peru, recently graduated physicians participate in the Servicio Rural Urbano Marginal en Salud (Rural-Urban Marginal Health Service [SERUMS], in Spanish), which involves working in rural areas of Peru for 1 year [1]. This kind of initiative undertaken in several countries in Latin America has been shown to be effective in attracting the participation of health professionals in these areas, enhancing the distribution of and access to health services in underserved populations [2]. Although SERUMS allows newly graduated physicians their first work experience in a primary care setting, participation in this service raises several challenges for these professionals, such as limited logistical and human resources in rural health facilities [3] and physicians’ lack of knowledge about the sociocultural factors [4] of the population they serve [5,6]. This situation, observed in various countries, has shown to affect doctors practicing in rural areas, reducing their job satisfaction and levels of well-being [7].

In line with this evidence, SERUMS physicians in Peru have been identified as a population with a remarkably high prevalence of mental health problems, even higher than that observed in the general population [5,6]. In addition, the COVID-19 pandemic is likely to have exacerbated this problem, as the prevalence of disorders, such as burnout, anxiety, depression, and alcohol use, among health care workers has increased [8-12]. In alignment with this, the Universidad Nacional Mayor de San Marcos (UNMSM) introduced a program to offer remote accompaniment and orientation to support mental health for physicians serving in rural areas during the SERUMS period.

In general, the use of telemedicine-based interventions has been shown to have positive effects on the well-being of physicians [13] and has proven to be effective in managing mental health problems among rural populations [14]. However, there is limited evidence regarding the effects of such interventions among SERUMS physicians in rural areas where the implementation of other remote interventions has proven to have a limited reach [15]. Therefore, our study aimed to describe the characteristics and perceptions of recently graduated physicians who work in rural areas of Peru toward a telehealth intervention to provide remote orientation and accompaniment in mental health.

## Methods

### Study Design and Context

We conducted a mixed methods study using a sequential explanatory design, which is a recommended approach that sequentially integrates quantitative cross-sectional and qualitative data for evaluating telehealth interventions [16].

### Study Population

Participants were SERUMS physicians enrolled in the Mental Health Accompaniment Program (MHAP) of the UNMSM. We used a list of 2021 medical graduates from the university who were scheduled to join SERUMS in 2022. To recruit participants, we implemented a process that involved distributing invitations via WhatsApp messages and phone calls. Approximately 1 month after starting their SERUMS, stakeholders who accepted the invitation received a questionnaire of mental problems screened. This questionnaire included an informed consent form.

### The Mental Health Accompaniment Program

The MHAP was designed and implemented by the Faculty of Medicine of the UNMSM from August 2022 to February 2023. Its main objective was to provide remote orientation and accompaniment on mental health for physicians graduating from this university who were going to participate in SERUMS. The program used a virtual platform and involved a team of trained personnel comprising psychiatrists, psychologists, and trained general physicians responsible for providing brief intervention and suicide prevention services. A designated coordinator team was responsible for supervising all program activities to ensure its smooth and effective implementation.

The MHAP included 4 services: evaluation and screening, self-help, brief intervention, and suicide prevention.

#### Evaluation and Screening Service

During the first months of SERUMS, all registered physicians at the MHAP were required to complete virtual questionnaires to assess the presence and severity of conditions such as depression, anxiety, alcohol consumption, and suicidal risk. Based on their results, participants were provided with appropriate care options tailored to their mental health status. In this process, specific criteria were established to determine the severity of cases and the history of mental health problems:

- Severe cases: These included problems showing high scores on different psychometric scales: the 9-item Patient Health Questionnaire (PHQ-9), score≥20; 7-item Generalized Anxiety Disorder (GAD-7), score≥15; the Alcohol Use Disorders Identification Test (AUDIT), score≥20; and Ask Suicide-Screening Questions (ASQ), a positive response to any of the 5 questions, including question 5, which was considered an emergency, or refusal to answer.
- History of mental health problems: This referred to any mental health problem that had required prior pharmacological treatment.
- Treatment received: This included any mental health problem that had already received the recommended treatment previously.

#### Self-Help Service

All physicians enrolled in the program had low severity of mental health problems and were offered access to the self-help service that includes the use of the Kusikuy website (Multimedia Appendix 1), an exclusive platform designed for the program that offers psychoeducational material on mental health, including self-help tools that allowed SERUMS physicians to self-manage issues, such as depression, anxiety, stress, problematic alcohol consumption, and fear of COVID-19

#### Brief Intervention Service

The brief intervention [17,18] is a transdiagnostic strategy based on the focused acceptance and commitment therapy developed by Stroshal and Robinson [19] that has the characteristics of being time limited, has been shown to be effective, and is short term. In addition, it aims to promote psychological flexibility in people through dialogue focusing on 3 pillars (as the authors call it): openness, awareness, and commitment.

The intervention process involves (1) framing, where therapists presents themselves as having an attitude of validation; (2) an assessment, which consists of a contextual interview, identification of the level of functionality, and the 3 pillars (in the commitment pillar, personal values are identified); and (3) the intervention itself, which includes behavioral experiments through a commitment to change aligned with the identified personal values [19].

The brief intervention varies from 2 to 4 sessions of approximately 20-40 minutes per participant. The first session develops the entire process, and subsequent sessions are a reinforcement of the observation of the 3 pillars and analysis of the results of the behavioral experiments, with emphasis on behaviors that promote or limit behavioral change [20]. The therapists were mental health professionals who participated in training on contextual therapies training by a certified professional, including acceptance and commitment therapy.

#### Suicide Prevention Service

SERUMS physicians who exhibited any level of suicide risk were provided with the suicide prevention service. A specialized psychiatrist contacted the physicians through instant messaging or phone calls to provide immediate assistance and guidance for managing their condition and facilitate prompt attention.

The provision of the brief intervention Service and suicide prevention services included several appointments between mental health professionals and SERUMS physicians for ongoing support and guidance until the physicians’ condition improved or was effectively managed. Severe cases receive additional counseling to continue meeting a specialist in a health care facility.

### Variables and Instruments

For the quantitative phase, we used data about the sociodemographic characteristics of physicians, such as gender and age, as well as the characteristics of the health care facilities where they worked, such as the region, level of complexity, and originating institution, collected during the program through virtual forms. This also included the evaluation of the mental health conditions of physicians through validated questionnaires, such as depression (PHQ-9), anxiety (GAD-7), alcohol use (AUDIT), and suicide screening (ASQ) [21–24]; the application of these instruments lasted for approximately 20-25 minutes. The presence of a history of mental health issues, including current or previous presence of mental health problems or the use of psychiatric medication, was also reported. Furthermore, the total number of participants and sessions conducted in the counseling and suicide prevention services was evaluated.

For the qualitative phase, the study had a qualitative inductive phenomenological design because of its usefulness in understanding and interpreting people’s experiences and meanings. This design is considered suitable for locating the meanings that people give to the events, processes, and structures of their lives and their perceptions, presuppositions, and assumptions. The use of this method allows us to know in depth their perceptions, appreciating their particularities, entering into the phenomenological explanations that quantitative research does not reach to analyze [21].

In-depth interviews were conducted using a semistructured interview guide. Physicians who initially agreed to participate in the MHAP were interviewed over the phone. All the interviewed physicians were asked about their perceptions of (1) the current mental health situation in SERUMS physicians and (2) the importance of developing a mental health intervention for this population. Specifically, those physicians who received some of the services of the MHAP were asked about their perceptions of and recommendations for these services, and those physicians who did not receive any service were asked about their reasons for not using the program. Interviews lasted approximately 15 minutes each and were audio-recorded and transcribed for subsequent analysis.

### Data Analysis

For quantitative analysis, we conducted a descriptive analysis using RStudio version 4.2.3 (Posit). Numeric variables were summarized using means (SDs), while categorial variables were presented in tables showing frequencies and percentages. For qualitative analysis, we conducted a thematic analysis using ATLAS.ti 9.0 software (ATLAS.ti Scientific Software Development GmbH). This involved coding the content according to the objectives of the interviews and subsequently linking those codes to generate categories that reflected the interviewees’ perceptions. Finally, quantitative and qualitative results were evaluated and interpreted together in order to draw conclusions that addressed the stated objectives.

### Ethical Considerations

The study was approved by the Research Ethics Committee of the Faculty of Medicine at the UNMSM (approval number 0172). Compliance with all relevant ethical approvals and established procedures for research involving human subjects was ensured. All participants in the study provided their informed consent to participate. No form of financial compensation was offered to participants for their involvement in this study. Furthermore, measures were implemented to analyze the data anonymously, ensuring the privacy and confidentiality of the collected information.

## Results

### Quantitative Results

**Figure 1:**
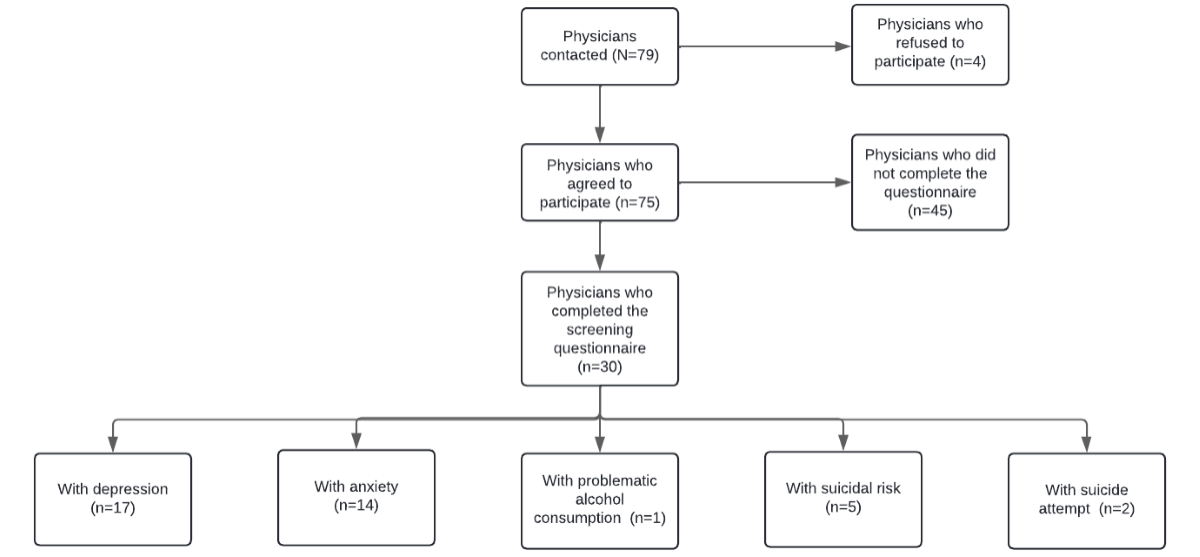
Flowchart of SERUMS physicians' involvement in the MHAP. MHAP: Mental Health Accompaniment Program; SERUMS: Servicio Rural Urbano Marginal en Salud (Rural-Urban Marginal Health Service).

Of the 116 physicians who graduated from the UNMSM and participated in SERUMS, 79 (68.1%) were contactable and 75 (94.9%) agreed to participate in the MHAP (see Figure 1). By the end of this intervention, 30 (41.6%) of the enrolled physicians ended up using at least 1 of the services offered in the MHAP (see Table 1). The mean age of these 30 physicians was 26.8 (SD 1.9) years, and 17 (56.7%) were women. Concerning their health care facilities during SERUMS, 15 (50%) belonged to category I-2, and 28 (93.3%) belonged to the Peruvian Ministry of Health (MINSA). Most of these facilities were located in Huanuco (n=7, 23.3%) and Huancavelica (n=6, 20%).

All the 9 (30%) evaluated physicians who exhibited moderate (n=7, 77.8%) or high (n=2, 22.2%) severity in at least 1 of the evaluated conditions agreed to receiving the brief intervention service over the phone or via video calls from a member of the MHAP health staff. The 2 (22.2%) participants categorized as “high severity” expressed suicidal intent during the program. Their evaluations were conducted by a psychiatrist, who confirmed the presence of a clinical syndrome in these physicians. Upon concluding their follow-up in the program, both physicians expressed a preference to continue treatment with the program’s psychiatrist, opting not to seek care in other institutions, such as the social security system in Peru (EsSalud), through which they are insured via their employment. During the follow-up period, no incidents of relapse (high risk) or worsening (moderate risk) were detected in the intervened cases.

**Table 1:** Mental health–screening results by sociodemographic and mental health history characteristics among SERUMS^a^ physicians enrolled in the MHAP^b,c^.

Characteristics		Depression				Anxiety			Alcoholic risk			Suicide risk			Any mental disease				Total (N=30)
		Any (n=17)	None (n=13)			Any (n=14)	None (n=16)		Any (n=4)	None (n=26)		Any (n=5)	None (n=25)		Any (n=21)	None (n=9)			
**Gender, n (%)**																			
	Female	11 (64.7)	6 (46.2)		10 (71.4)		7 (43.8)	2 (50.0)		15 (57.7)	4 (80.0)		13 (52.0)	13 (61.9)		4 (44.4)		17 (56.7)	
	Male	6 (35.3)	7 (53.8)		4 (28.6)		9 (56.2)	2 (50.0)		11 (42.3)	1 (20.0)		12 (48.0)	8 (38.1)		5 (55.6)		13 (43.3)	
Age (years), mean (SD)		26.9 (1.60)	26.5 (2.30)	27.1 (1.92)			26.4 (1.90)	25.5 (3.32)		27.0 (1.61)	27.2 (2.28)		26.7 (1.86)	26.5 (2.02)		27.3 (1.58)	26.8 (1.91)		
**History of mental disorder, n (%)**																			
	Anxiety and depression	1 (5.9)	0		1 (7.1)		0	0		1 (3.8)	0		1 (4.0)	1 (4.8)		0		1 (3.3)	
	Anxiety	4 (23.5)	0		3 (21.4)		1 (6.3)	1 (25.0)		3 (11.5)	1 (20.0)		3 (12.0)	4 (19.0)		0		4 (13.3)	
	Depression	5 (29.4)	0		3 (21.4)		2 (12.5)	0		5 (19.2)	1 (20.0)		4 (16.0)	5 (23.8)		0		5 (16.7)	
	Suicide attempt	1 (5.9)	0		1 (7.1)		0	1 (25.0)		0	1 (20.0)		0	1 (4.8)		0		1 (3.3)	
	No history of mental disorder	6 (35.3)	13 (100.0)		6 (42.9)		13 (81.3)	2 (50.0)		17 (65.4)	2 (40.0)		17 (68.0)	10 (47.6)		9 (100.0)		19 (63.3)	
**Mental health treatment, n (%)^d^**																			
	No therapy	4 (36.4)	0		3 (37.5)		1 (33.3)	2 (100.0)		2 (22.2)	2 (66.7)		2 (25.0)	4 (36.4)		0		4 (36.4)	
	Pharmacological and psychological therapy	3 (27.3)	0		1 (12.5)		2 (66.7)	0		3 (33.3)	0		3 (37.5)	3 (27.3)		0		3 (27.3)	
	Psychological therapy	4 (36.4)	0		4 (50.0)		0	0		4 (44.4)	1 (33.3)		3 (37.5)	4 (36.4)		0		4 (36.4)	

^a^SERUMS: Servicio Rural Urbano Marginal en Salud (Rural-Urban Marginal Health Service).

^b^MHAP: Mental Health Accompaniment Program.

^c^The sum of percentages for some subheadings might be less than or more than 100 because of rounding.

^d^Only those who reported a history of mental disease.

### Qualitative Results

For the qualitative phase, we interviewed 8 (26.7%) of the 30 participants, 4 (50%) who used the program and 4 (505) who did not. To estimate the number of selected physicians in this phase, we used the saturation technique, which incorporates participants until their responses no longer provide further information regarding the objectives of the interview.

#### Mental Health Situation in SERUMS Physicians

Participants reported experiencing behavioral and mood changes, increased anxiety, nervousness, sadness, difficulty sleeping, headaches, and adjustment disorders during the initial months of SERUMS. They also noted that several colleagues underwent similar experiences, including episodes of depression.

I believe that I experienced a mental health problem, perhaps it could be an adjustment disorder. I realized it because normally I am cheerful and always feel like doing things, or at least most of the time I feel like doing things, but the joy decreased, I felt less joyful and less motivated to do things.

Participant 3

Several of my colleagues with whom I have a close relationship from university also had mental health problems...one of my colleagues was quite depressed, I don’t know to what extent, but he was definitely quite depressed, and another one of my colleagues also experienced depression, and that is what we see the most among SERUMS physicians.

Participant 1

#### Importance of Mental Health Interventions

Participants emphasized the critical nature of mental health interventions for physicians in SERUMS. They highlighted the necessity of guidance for all SERUMS health care professionals who consistently face stressful situations. These stressors are motivated by challenges arising from adapting to new environments, distance from family and friends, internet connectivity issues limiting communication with loved ones, and difficulties in their work environment. Consequently, they require tools to cope with such events effectively.

Well, I believe it is fundamental, it is essential for a health care professional, regardless of whether they are going through a specific problem. I think we all should have mental health support because, in SERUMS, for example, we face different things. We are not in Lima, but in an environment that is not comfortable, so we face different adversities, and we need guidance that helps us stay calmer and cope with the situation.

Participant 4

For example, I am from Piura. I finished high school and then came to Lima alone. I even went abroad for a month for an exchange program. And when my SERUMS came, I thought it wouldn't affect me, everything would be fine, but it is very different...perhaps because you have greater responsibility, and every action you take will weigh on and affect others. That is why you experience a lot of stress. So, I believe providing mental health support for us is very important, and I appreciate this initiative.

Participant 3

#### Perception of the Mental Health Program

SERUMS physicians indicated that the resources provided in the MHAP were significantly beneficial, expressing satisfaction with the services received. From their perspective, interactions with the mental health team were highly professional, and they established a good relationship as they were provided with a trusting, listening, and guiding space, enabling them to cope with their challenges. Regarding the resources on the Kusikuy website, they mentioned having difficulties accessing the page due to an unstable internet connection. Nonetheless, they found the content they could access useful.

In my experience so far, everything has been good. I am quite satisfied with the service they have provided me, so everything is fine, and it has helped me a lot, to be honest.

Participant 4

Very good, really. It supports you a lot, knows how to listen to you, knows how to guide you…excellent!

Participant 2

Very good service with the doctor, it gave me confidence...I think it has been a good initiative on your part to provide this mental health service.

Participant 3

I took a general look at it, but I didn’t use it much. From what I could see, it seemed like an interesting website with plenty of resources, but I couldn’t explore it further due to internet problems, and when I had a good internet connection, I forgot about it.

Participant 1

#### Reasons for Not Using the Program

Some physicians did not engage with the program, even though they reported experiencing emotionally distressing situations and mental health issues. Among the reasons for not accessing the program, many physicians mentioned not having sufficient time to properly inform themselves about the program, or they received information during their working hours, which led to postponing reading those messages and eventually forgetting to respond. This resulted in them being uninformed about the program and, therefore, not using it.

Basically, it is because I didn’t have time. Being a head at my health center, I was busy almost all the time. At one point, I did consider seeking help from the service, but I didn’t have the time.

Participant 1

I think the main reason is that, for example, when I wanted to enroll or get information about the program, they would call or send messages during working hours, which is actually an inconvenient time because you are attending to patients and can't respond at that moment. Then, I would forget to reply, and that is how it went...I would prefer them to call me outside of working hours, where I could attend to them better and get more information.

Participant 3

Another reason mentioned by participants is that although they experienced feelings of discomfort, they chose to ignore those sensations and continue with their routine. Alternatively, they mentioned using their personal resources, such as maintaining stronger connections with their support network and engaging in distracting and entertaining activities, among others. Therefore, they did not feel the need to use the service, as they used their personal tools.

I wanted to use the mental health service; I was interested in the program since the SERUMS experience was quite challenging for me...it is just that I think I preferred to ignore my discomfort and continue with my routine. I buried all my distress and sadness to focus on my other tasks.

Participant 2

While the first few months were difficult in SERUMS, I feel like I didn't require the service, because it was an adaptation process that was mine to handle. I chose to call my friends, be with my family, study for the residency exam, watch TV series, and that is how the days passed. I adapted with my own resources.

Participant 4

Other reasons mentioned were related to the lack of publicity for the program. Not everyone was clear about the services provided in the program, and they were unaware of the existence of the Kusikuy website, which they believed could have been a great help. They also expressed the hope that the program would include other tools, such as workshops on communication and leadership, among other topics, as they are necessary and useful for SERUMS, and could also be attractive for encouraging participation in the program.

I think they need to improve the advertising because I was unaware of the website, which would have been very helpful due to all the materials they had, but I didn’t know about it. So, one of the reasons why we didn’t use their resources is because we didn’t know about them.

Participant 3

In addition to the services I knew they would provide, it would also be useful if they organized workshops on communication tools, leadership models, improving the work environment, and even workshops on how to interact better with patients. I think it would also be appealing to the SERUMS participants to encourage us to use the program.

Participant 2

#### Recommendations for Improving the Mental Health Program

Among the recommendations for the MHAP, SERUMS physicians suggested increasing the frequency of sessions with mental health professionals, as they are currently limited to once a month. They also deemed it necessary to reduce the time between completing the initial survey and the first session with the mental health professionals, as it took between 3 and 4 weeks before receiving communication. Additionally, they expressed a preference for having an application instead of the Kusikuy website, due to the difficulties they experienced in accessing it. They also wished to have the option to download resources and use them offline.

Beyond the services provided in the MHAP, participants expressed their desire to have support groups composed of other serum physicians and led by mental health professionals. They requested the inclusion of more resources related to stress management, anxiety, study techniques, personality tests, work environments, and conflict resolution among colleagues, and they wanted those resources to be provided through WhatsApp.

Perhaps the sessions with the doctor could be a little more frequent and not just once a month. I know it is due to the teachers’ time and that it is free, but I would like it to be more than once a month. Apart from that, I have no criticism of the program; I am satisfied with this initiative.

Participant 2

Regarding the website you designed, I think it could be an application or through WhatsApp or whatever it may be, but it should have the ability to download videos or use videos offline...without relying so much on the internet.

Participant 3

## Discussion

### Principal Findings

The results of the study suggest that the implementation of the MHAP is effective in identifying and managing mental health conditions in newly graduated physicians working in rural areas of Peru in the context of SERUMS. Although we found no evidence of digital mental health interventions for these professionals, our findings are consistent with other studies highlighting the usefulness of digital tools in identifying and treating mental health problems [22]. In addition, it is suggested that interventions aimed at addressing COVID-19–related mental health issues among health care workers may improve coping skills and reduce symptoms, such as anxiety, depression, and posttraumatic stress disorder [23], as well as problematic alcohol use [24].

Despite the initial interest of SERUMS physicians in the MHAP, their real commitment diminished once they were assessed in the initial part of the program. This phenomenon could be due to the current tendency seen in people to ignore mental health concerns in their daily routine and the stigma associated with seeking help [25-27]. Among the factors that we found to limit their participation included a preference for using personal resources to address mental health issues and a lack of time or connectivity to participate in online activities that are in concordance with evidence reported elsewhere [28-32]. These findings highlight the complexity of addressing mental health difficulties and underscore the importance of considering various factors, from beliefs and stigma to resource availability and satisfaction with proposed interventions, to promote a more effective approach.

Another noteworthy finding of our study was the high proportion (around 50%) of SERUMS physicians who reported experiencing depression or anxiety, and 5 of them even expressed suicidal thoughts, indicating a significant problem in their mental health compared to previous studies [4,5]. Despite being noticeable, this result should be interpreted cautiously as it may overestimate the true prevalence, because it was based only on physicians who agreed to be evaluated in the program. Nevertheless, the fact that there is a significant frequency of SERUMS physicians with mental health problems highlights the need to address this problem. The inherent vulnerability to SERUMS conditions and concern about the increase in mental health problems among undergraduate medical students [33] leads to an increased risk of mental health problems among physicians. It is crucial to address this concern by deploying more health staff or teams to meet the growing demand for psychological support.

SERUMS physicians with moderate and severe levels of mental health issues demonstrated good engagement with the services of the program, participating in all sessions with specialists. Similar findings have been observed in other studies, where mental health status influenced the interest in and the use of digital mental health interventions [25,34,35]. It is important to note that this engagement was more common within the program than in the usual care channels provided to SERUMS physicians through social security hospitals. In addition, although SERUMS physicians had a mostly positive perception of MHAP services, some experienced difficulties with virtual tools, such as access to the Kusikuy website, which has been identified in other research as a barrier to participation in similar interventions [25,27,35]. Therefore, it is essential to ensure that broadcasting and distance education services are available on platforms with low connectivity requirements or are easily accessible through mobile devices, which are the most used by these professionals.

### Limitations and Strengths

Although these results are significant, it is important to recognize certain limitations. The interviews were conducted at the end of the SERUMS year, which increased the possibility of recall bias and could have affected the accuracy of the information collected. In addition, it is important to consider the influence of social desirability bias on our results, given the stigma still associated with mental health problems, so participants may have tended to underestimate or minimize their experiences. This bias could have influenced the reported incidence rates of mental health problems among SERUMS physicians, which could have led to an underestimation of the true prevalence.

Despite these limitations, our study provides important insights into the high incidence of mental health problems among SERUMS physicians and the potential of digital mental health interventions to close the gap in access to mental health services. In addition, the MHAP demonstrated several strengths that have shown positive outcomes in the mental health of SERUMS physicians, such as timely identification, participation in services, and satisfaction with them.

### Conclusion

The MHAP has proven to be a valuable intervention for addressing mental health issues among SERUMS physicians in rural areas of Peru, demonstrating its effectiveness in both identifying and managing mental health conditions in this population. Despite the good participation and reception of the services by the participants, challenges were faced in terms of access and participation. The identification of previous mental health problems among physicians and their willingness to participate in the program highlights the importance of mental health interventions in this context. However, barriers related to the lack of time, the stigma associated with seeking help, and a preference for personal resources underscore the need to improve program accessibility and promotion. Some recommendations that can be included are diversifying the resources offered, raising awareness about the importance of self-care, and promoting support spaces among participating physicians. This will maximize the impact and benefits of the program for SERUMS physicians in Peru.

## Appendix

Multimedia Appendix 1 Kusikuy website interface.

Multimedia Appendix 2 Scores of mental health outcomes in MHAP. AUDIT: Alcohol Use Disorders Identification Test; GAD-7: 7-item Generalized Anxiety Disorder; MHAP: Mental Health Accompaniment Program; PHQ-9: 9-item Patient Health Questionnaire.

## Abbreviations

ASQ: Ask Suicide-Screening Questions
AUDIT: Alcohol Use Disorders Identification Test
GAD-7: 7-item Generalized Anxiety Disorder
MHAP: Mental Health Accompaniment Program
PHQ-9: Patient Health Questionnaire
SERUMS: Servicio Rural Urbano Marginal en Salud (Rural-Urban Marginal Health Service)
UNMSM: Universidad Nacional Mayor de San Marcos

## Data Availability

The data sets analyzed during the study are not publicly available as they are part of confidential information of Servicio Rural Urbano Marginal en Salud (SERUMS) physicians involved in the Mental Health Accompaniment Program of the Universidad Nacional Mayor de San Marcos. However, these could be anonymized and shared by the corresponding author upon reasonable request.
