# Supplementary figures and images for: Assessment of a Pilot Program for Remote Support on Mental Health for Young Physicians in Rural Settings in Peru: Mixed Methods Study
Source: JMIR Form Res. 2024 Sep 10;8:e54005. doi: 10.2196/54005 (PMC11422723; doi:10.2196/54005)

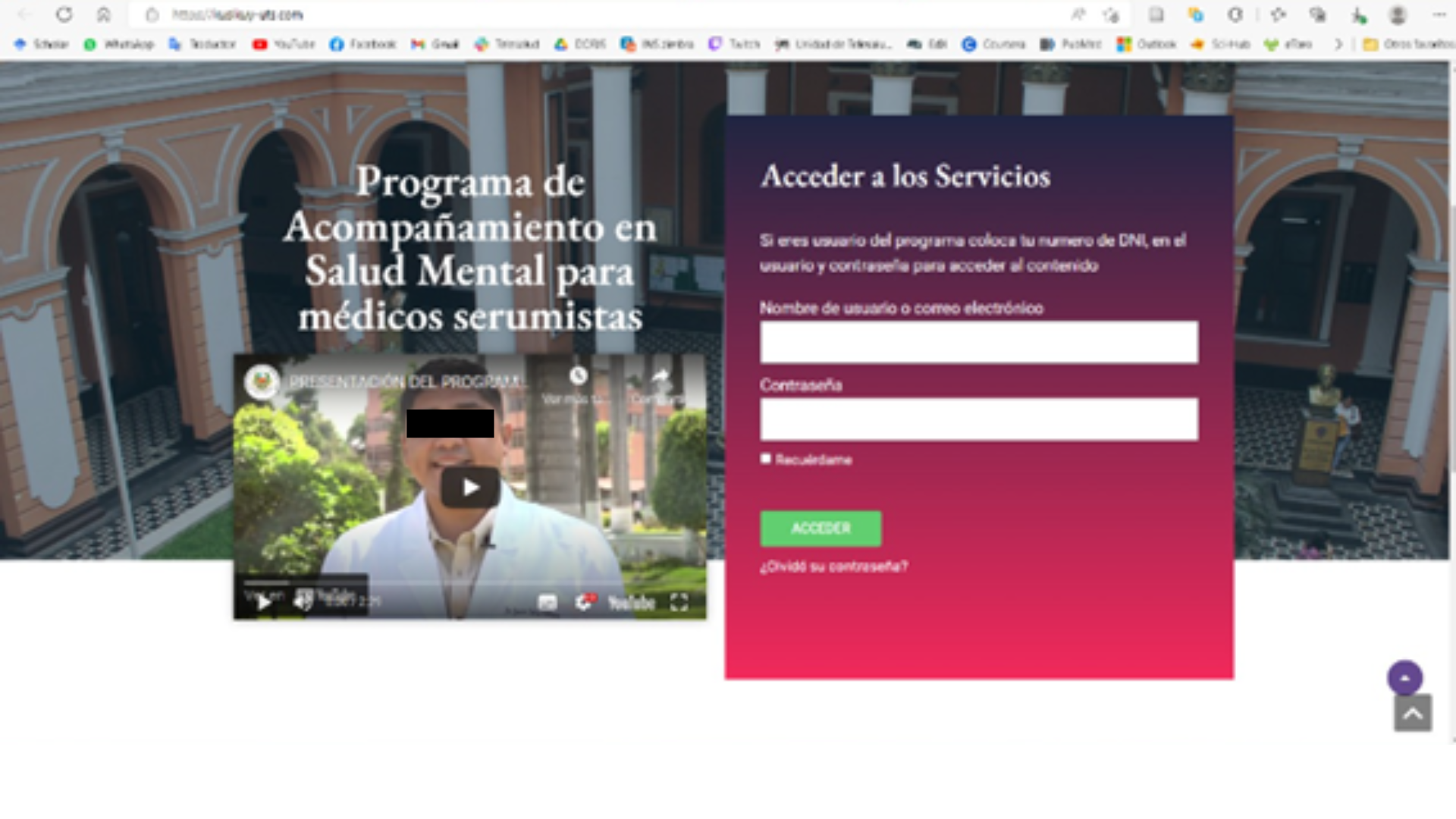

Supplement: Multimedia Appendix 1 [file formative_v8i1e54005_app1.png]

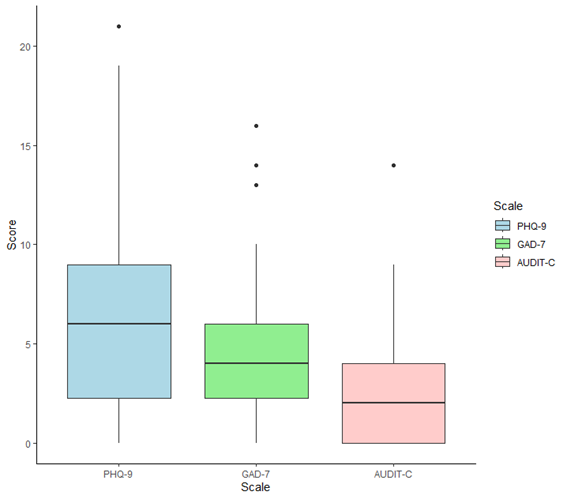

Supplement: Multimedia Appendix 2 [file formative_v8i1e54005_app2.png]
